# Evaluation of the Cytotoxic and Antiviral Effects of Small Molecules Selected by In Silico Studies as Inhibitors of SARS-CoV-2 Cell Entry

**DOI:** 10.3390/molecules28207204

**Published:** 2023-10-21

**Authors:** Francisca Carvalhal, Ana Cristina Magalhães, Rita Rebelo, Andreia Palmeira, Diana I. S. P. Resende, Fernando Durães, Miguel Maia, Cristina P. R. Xavier, Luísa Pereira, Emília Sousa, Marta Correia-da-Silva, M. Helena Vasconcelos

**Affiliations:** 1 FFUP—Faculty of Pharmacy, University of Porto, 4050-313 Porto, Portugalrrebelo@ipatimup.pt (R.R.); apalmeira@ff.up.pt (A.P.); dresende@ff.up.pt (D.I.S.P.R.); fduraes5@gmail.com (F.D.); miguelmaia2@gmail.com (M.M.); esousa@ff.up.pt (E.S.); 2 CIIMAR—Interdisciplinary Centre of Marine and Environmental Research, 4408-208 Matosinhos, Portugal; 3 i3S—Instituto de Investigação e Inovação em Saúde, University of Porto, 4200-135 Porto, Portugal; acmagalhaes@i3s.up.pt (A.C.M.); cristinax@ipatimup.pt (C.P.R.X.); luisap@i3s.up.pt (L.P.); 4 IPATIMUP—Institute of Molecular Pathology and Immunology, University of Porto, 4200-135 Porto, Portugal

**Keywords:** SARS-CoV-2 receptor-binding domain, spike protein, ACE2, antiviral, small molecules, virtual screening

## Abstract

The spike protein of the severe acute respiratory syndrome coronavirus 2 (SARS-CoV-2) relies on host cell surface glycans to facilitate interaction with the angiotensin-converting enzyme 2 (ACE-2) receptor. This interaction between ACE2 and the spike protein is a gateway for the virus to enter host cells and may be targeted by antiviral drugs to inhibit viral infection. Therefore, targeting the interaction between these two proteins is an interesting strategy to prevent SARS-CoV-2 infection. A library of glycan mimetics and derivatives was selected for a virtual screening performed against both ACE2 and spike proteins. Subsequently, in vitro assays were performed on eleven of the most promising in silico compounds to evaluate: (i) their efficacy in inhibiting cell infection by SARS-CoV-2 (using the Vero CCL-81 cell line as a model), (ii) their impact on ACE2 expression (in the Vero CCL-81 and MDA-MB-231 cell lines), and (iii) their cytotoxicity in a human lung cell line (A549). We identified five synthetic compounds with the potential to block SARS-CoV-2 infection, three of them without relevant toxicity in human lung cells. Xanthene **1** stood out as the most promising anti-SARS-CoV-2 agent, inhibiting viral infection and viral replication in Vero CCL-81 cells, without causing cytotoxicity to human lung cells.

## 1. Introduction

Coronavirus disease 2019 (COVID-19) is a viral respiratory infection that emerged in Wuhan, China, at the end of 2019, as a novel zoonotic disease caused by the severe acute respiratory syndrome coronavirus 2 (SARS-CoV-2), and which rapidly spread worldwide. In March 2020, the World Health Organization (WHO) declared this infectious disease a pandemic [1]. As of October 2023, SARS-CoV-2 has infected 771,191,203 people and caused 6,961,014 deaths globally, according to WHO (https://covid19.who.int, accessed on 14 October 2023). The emergence of new viral variants and the limitations of the existing vaccines and treatment options highlight the need for new therapies.

SARS-CoV-2 is an enveloped, positive-sense, single-stranded RNA virus (+ssRNA) with a crown-like appearance given by the presence of spike proteins in the viral membrane [2,3]. The main cellular receptor for this virus is ACE2, but other potential host receptors include neuropilin 1, dipeptidyl peptidase 4, glucose-regulated protein-78, and cluster of differentiation 147 [2]. The attachment and fusion of the viral particle to the host cell membrane are mediated by the spike protein, which is formed by two noncovalently associated subunits, S1 and S2. The first one (S1) binds to the host cell receptor ACE2 and the second one (S2) attaches the spike protein to the membrane and integrates a fusion peptide to mediate the fusion between viral and cellular membranes [4]. The SARS-CoV-2 receptor-binding domain (RBD) present in the viral spike protein shares structural and sequential similarities with other coronaviruses, which are essential for ACE2 binding. Thus, targeting the conserved epitopes in the RBD might be a promising strategy to develop cross-reactive therapeutic agents against diverse coronaviruses. 

However, ACE2 is necessary for vital host functions, such as blood pressure and kidney function, regulation of the renin-angiotensin-aldosterone system, and processing angiotensin-2, consequently, mediating vasoconstriction, pro-fibrotic, and pro-inflammatory processes [4]. Therefore, some studies suggest that the downregulation of ACE2 levels due to SARS-CoV-2 infection might increase COVID-19 severity, leading to acute lung injury, which presents an additional challenge to the cytotoxicity of the virus itself [5,6]. Blocking the binding of spike protein to ACE2 is a logical and promising therapeutic strategy [7,8,9,10], but it is important to preserve ACE2 physiological levels. 

Sulfated glycosaminoglycans (GAGs) displayed at the surface of host cells, like Heparan Sulfate (HS), have been shown to play an important role in the attachment of enveloped viruses to the cell surface, namely of coronaviruses, herpes simplex virus, cytomegalovirus, dengue virus, and hepatitis B virus [11]. Due to the heavily sulfated GAG chains, HS possess a global negative charge that allows for electrostatic interactions with the basic residues found on viral surface glycoproteins. Viruses exploit these weak interactions to enhance their concentration at the cell surface, thereby increasing their likelihood of binding to a more specific entry receptor, triggering viral infection [12]. The spike glycoprotein of SARS-CoV-2 is described as relying on host cell surface HS proteoglycans to facilitate interaction with the ACE2 receptor [13,14]. 

In this work, we hypothesized that our library of synthetic GAG mimetics and derivatives could act as inhibitors of the ACE2–spike interaction. To test this hypothesis, we performed a structure-based virtual screen of approximately 300 small molecules to identify the ones with the most affinity for the targets ACE2 and/or spike. Moreover, we provide evidence that five of the selected molecules inhibit SARS-CoV-2 infection in Vero-CCL81 cells and that three of them also inhibit viral replication. Further assays reveal that none of the five promising compounds affect ACE2 expression in infected cells and that three of them do not cause cytotoxicity in human lung cells. 

## 2. Results

### 2.1. Screening Studies Revealed Interaction of Several Synthetic Small Molecules with ACE2 Host Receptor and Spike Viral Protein

A structure-based virtual screening was carried out with an in-house library of 304 synthetic compounds containing diverse chemical structures (142 xanthones, 48 thioxanthones, 27 xanthenes, 10 bile acid derivatives, 16 coumarins, 14 flavonoids, and 47 polyphenolic compounds). Numerous compounds with potential to bind to the targets ACE2 and RBD of spike were identified. From this library, 223 molecules were found to have a lower free energy of binding (higher affinity) to ACE2 than the control [SARS-CoV-2 peptide (C480-C488), −6.3 kcal/mol], and 203 molecules were found to bind more tightly to the RBD of the spike protein than the control (all-trans retinoic acid, −6.7 kcal/mol). The ten compounds that presented the lowest negative docking scores for ACE and/or RBD of spike (highest affinity) were selected for functional studies (Table 1). Of these, three are xanthenes (**1** and **2** presenting bulky sulfonamides, and **3** showing symmetry); one is a bile acid derivative (**4**, with an amide derivative); and six are xanthones with bulky substituents (**5**–**10**, of which four are GAG-like xanthones with sulfated or hydroxylated sugar moieties, **6**–**9**; and two are aminated xanthones, **5** and **10**). Among the known drugs selected for this in silico study (darunavir, lopinavir, ponatinib, remdesivir, and ribavirin), ponatinib was the one with the best docking score (ACE2 = −8.3 kcal/mol; RBD of spike = −8.1 kcal/mol), and thus, it was included as a test compound in the functional assays.

**Figure 1:**
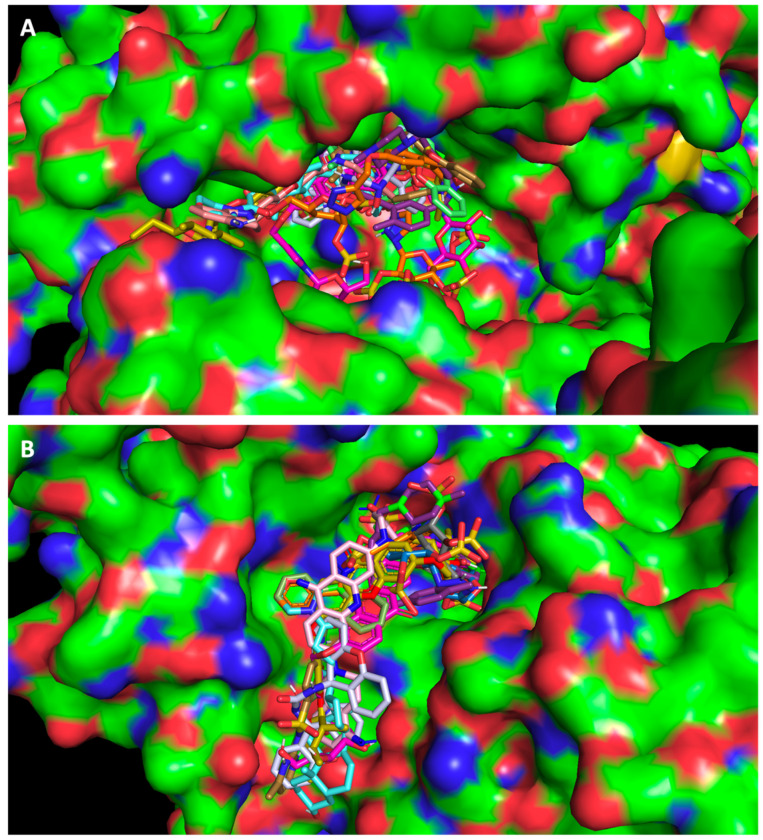
In silico virtual screening. Top-ranked selected test compounds docked into (**A**) ACE2 and (**B**) the RBD of the spike protein. The compounds selected from the in silico studies are represented with sticks in different colors. Crystal structures were obtained from the Protein Data Bank (ACE2, PDB code 6M18 [15] and the RBD of the spike protein, PDB code 6M0J [10]) and are represented as surface plots.

Using the PyMol program for visual inspection, the compounds were predicted to fit into the substrate binding site of ACE2 (Figure 1A) and the RBD of the spike protein (Figure 1B), and to bind in the same region as the positive controls. 

### 2.2. Five of the Selected Compounds Inhibited SARS-CoV-2 Viral Infection in Vero CCL-81 Cells

**Figure 2:**
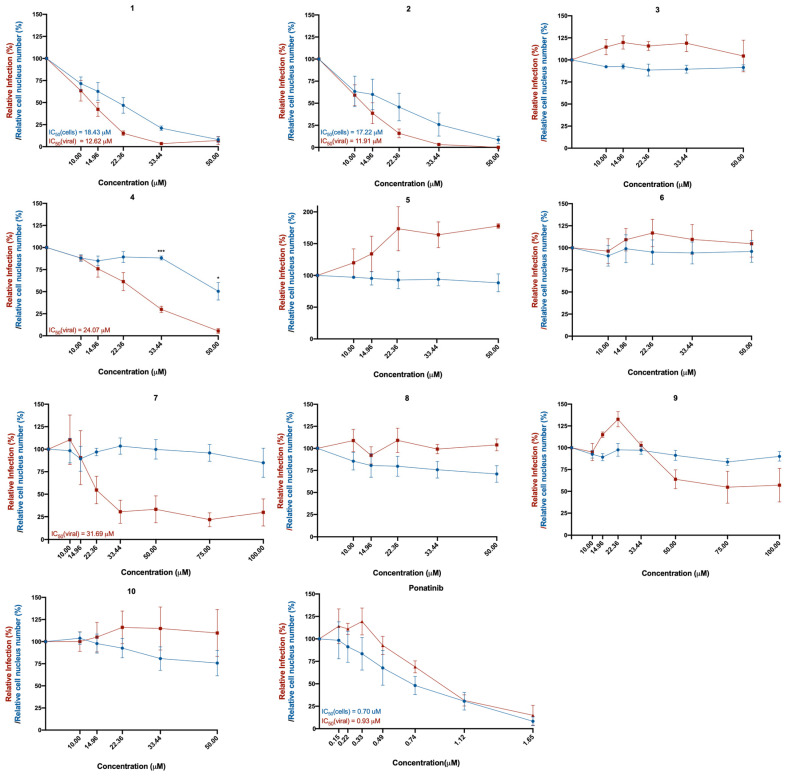
Effect of the tested compounds (**1**–**10**) in Vero CCL-81 cells infected with SARS-CoV-2 (MOI = 1). Vero CCL-81 cells were treated with the compounds (**1**–**10**) at the indicated concentrations and further infected with SARS-CoV-2 for 48 h. Cells were fixed with 4% PFA and stained for the SARS-CoV-2 spike protein. Immunofluorescence results were analyzed with the IN Cell Analyzer 2000. The relative number of viral infections is indicated by the red lines. The relative number of cell nuclei is indicated by the blue lines. Values are relative to control (infected cells without treatment). The respective IC_50_ values are indicated in the same colors as the concentration–response curves. Results represent the mean ± SEM of three independent experiments (each one performed in duplicate).

The Vero CCL-81 monkey epithelial kidney cell line was selected for cell infection, since these cells are known for being (i) susceptible to a wide range of viruses, including coronaviruses; (ii) easy to culture; and (iii) deficient in type I interferon, which facilitates viral replication and infection by SARS-CoV-2 [16,17]. These cells were treated with the in silico selected compounds **1**–**10** and with ponatinib, in concentrations ranging from 10 μM to 100 μM (or from 0.15 μM to 1.65 μM in the case of ponatinib, due to its known cytotoxicity) and subsequently infected with SARS-CoV-2 (MOI = 1). The effect of the compounds was investigated 48 h later, by analyzing the presence of the spike protein (viral marker) in treated cells. The results of cytotoxicity and infection levels are represented in Figure 2. The cytotoxicity curve (represented in blue) was calculated based on the number of cell nuclei (DAPI staining), while the relative infection curve (represented in red) was calculated based on the number of infected cells (the presence of the spike protein determined by immunofluorescence) relative to control (infected cells without treatment).

The toxicity of compounds in infected cells is slightly superior to the toxicity in non-infected cells (Supplementary Figure S1), due to the toxic effect of the infection itself [18]. However, treatment with compounds **3** and **4** showed significantly higher cytotoxicity in infected cells compared to non-infected cells at the same concentrations, suggesting that these compounds are affecting cytotoxicity in the infected cells. Curiously, compounds **3**, **4**, **7**, **9**, and **10** increased the relative cell number of non-infected cells.

**Figure 3:**
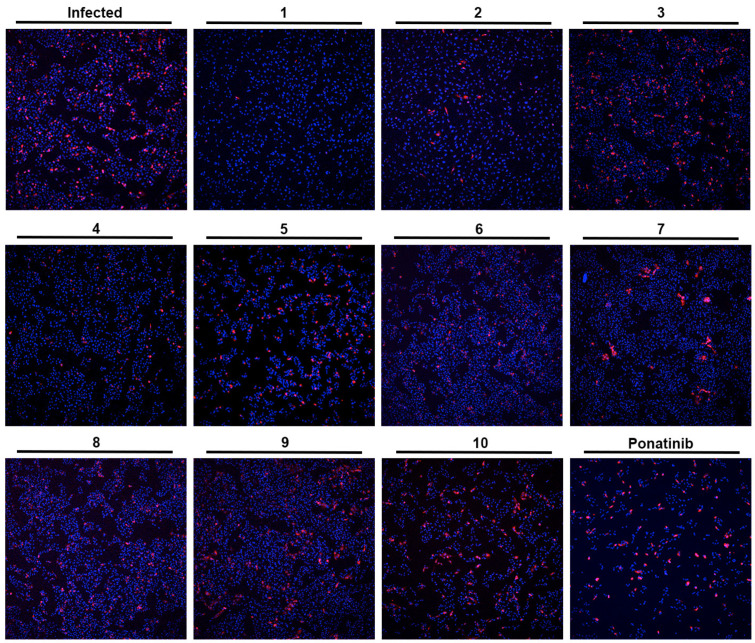
Effect of the tested compounds (**1**–**10**) in Vero CCL-81 cells infected with SARS-CoV-2 (MOI = 1). Representative images of the immunofluorescence staining of the SARS-CoV-2 spike protein (red) and nuclei (blue) of infected Vero CCL-81 cells, previously treated with 33 μM of the tested compounds (except Ponatinib: 0.49 μM; and compound **9**: 50 μM). Amplification of 10×.

As depicted in Figure 2 and Figure 3, treatment of Vero CCL-81 cells with sulfonamide xanthenes **1** and **2**, bile acid **4**, and glucosulfated xanthone **7** decreased the number of cells infected by SARS-CoV-2 when compared to control, presenting a half-maximal inhibitory concentration (IC_50_) between 11.91 μM and 31.69 μM. The glucosulfated xanthone **9** also reduced cell infection by SARS-CoV-2, but its IC_50_ was not reached at the tested concentrations. On the other hand, compounds **3**, **5**, **6**, **8**, **10**, and ponatinib (at the tested concentrations) did not inhibit cell infection.

Compounds with anti-SARS-CoV-2 activity should present low cytotoxicity at their effective antiviral concentration. This was not the case for ponatinib and xanthenes **1** and **2**, which presented some cytotoxicity in this model, as verified by a reduction in the number of cell nuclei (Figure 2, blue line). Nonetheless, considering that the Vero CCL-81 cell line is a non-human cell model, all the compounds that showed a reduction in the number of infected cells (**1**, **2**, **4**, **7** and **9**) were selected for further studies. Ponatinib was excluded since at the tested concentrations its toxicity was higher than its capacity to inhibit infection. 

### 2.3. The Compounds That Exhibited Inhibition of Viral Infection Were Also Predicted to Promisingly Interact with Residues of the Target Proteins

**Figure 4:**
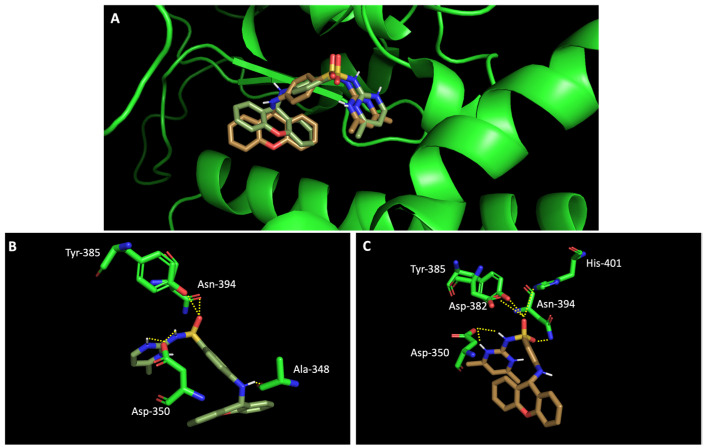
Molecular visualization of sulfonamide xanthenes **1** and **2** in ACE2. (**A**) General view of compounds **1** (green) and **2** (brown). (**B**) Interaction between compound **1** and ACE2. (**C**) Interaction between compound **2** and ACE2. Polar interactions are represented as yellow dashes. Residues involved on those interactions are labeled: Ala—alanine; Asn—asparagine; Asp—aspartic acid; His—histidine; Tyr—tyrosine.

**Figure 5:**
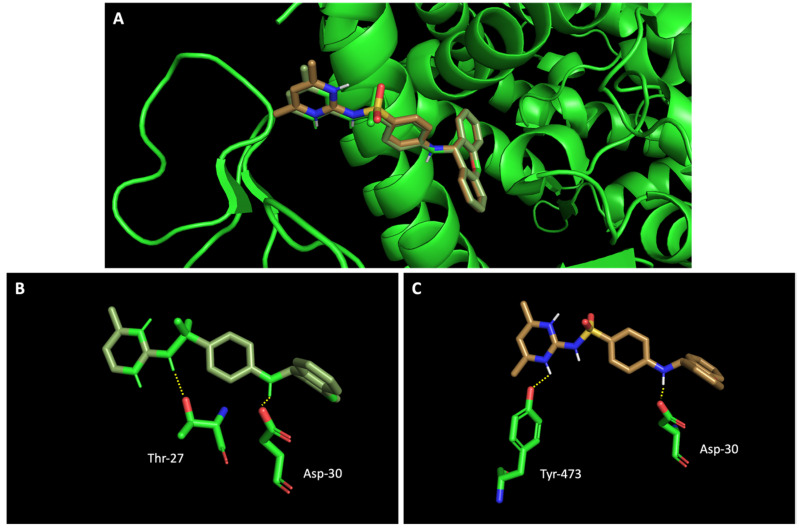
Molecular visualization of sulfonamide xanthenes **1** and **2** in the RBD of the spike protein. (**A**) General view of compounds **1** (green) and **2** (brown). (**B**) Interaction between compound **1** and the RBD of the spike protein. (**C**) Interaction between compound **2** and the RBD of the spike protein. Polar interactions are represented as yellow dashes. Residues involved on those interactions are labeled: Asp—aspartic acid; Thr—threonine; Tyr—tyrosine.

In silico studies concerning the interaction of compounds **1**, **2**, **4**, **7**, and **9** with ACE2 and the RBD of the spike protein were accomplished in order to predict which residues were involved in their interaction with the target. Upon first glance, it can be noted that the most favorable poses of the sulfonamide xanthenes **1** and **2** are identical to one another when interacting to ACE2 and the RBD of the spike protein, as can be seen in Figure 4A and Figure 5A, respectively. Both compounds seem to bind to the negatively charged amino acid Asp-350 and to the amino acids Tyr-385 and Asn-394 of ACE2 (Figure 4B,C), which can be justified by their structural similarity. Plus, compounds **1** and **2** were predicted to establish interaction with the negatively charged amino acid Asp-30 of the RBD of the spike protein (Figure 5B,C).

**Figure 6:**
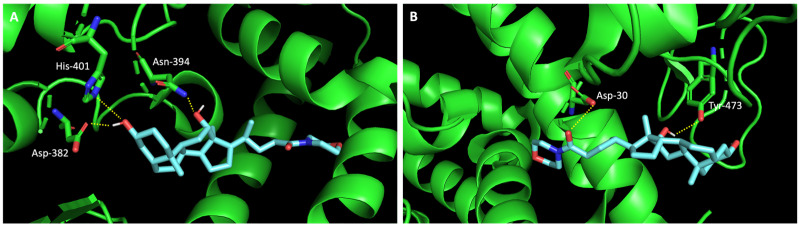
Molecular visualization of bile acid derivative **4** in (**A**) ACE2 and (**B**) the RBD of the spike protein. Polar interactions are represented as yellow dashes. Residues involved on those interactions are labeled: Asn—asparagine; Asp—aspartic acid; His—histidine; Tyr—tyrosine.

Additionally, studies on the most favorable pose of the steroid derivative **4** (Figure 6) show that it binds to the same amino acids of ACE2 and the RBD of the spike protein as sulfonamide xanthene **2**. 

**Figure 7:**
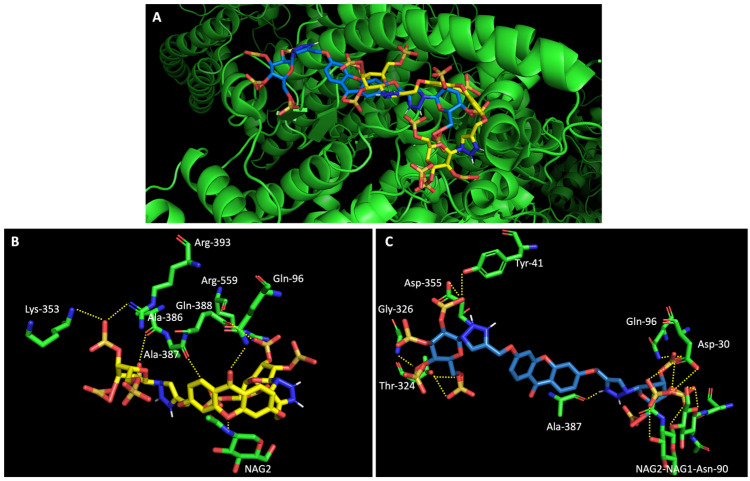
Molecular visualization of derivatives glucosulfated xanthone **7** and **9** in ACE2. (**A**) General view of compounds **7** (yellow) and **9** (blue). (**B**) Interaction between compound **7** and residues of ACE2. (**C**) Interaction between compound **9** and residues of ACE2. Polar interactions are represented as yellow dashes. Residues involved on those interactions are labeled: Ala—alanine; Arg—arginine; Asp—aspartic acid; Gln—glutamine; Gly—glycine; NAG—N-acetylglucosamine; Thr—threonine; Tyr—tyrosine.

On the other hand, a general view of the binding of glucosulfated xanthones **7** and **9** to ACE2 (Figure 7), shows that the derivatives share interactions with only two amino acids (Gln-96 and Ala-387). But it is also possible to observe that both derivatives interact with the glycan chain Asn90-linked NAG–NAG–β-_D_-mannose, which is described as essential for viral interaction with ACE2 [10,19]. Plus, docking studies with SARS-CoV-2 also predicted interactions with the residues Asp-30, Tyr-41, Asp 355, and Lys 353 of ACE2, suggesting the importance of these residues in the interaction with inhibitors [10]. 

**Figure 8:**
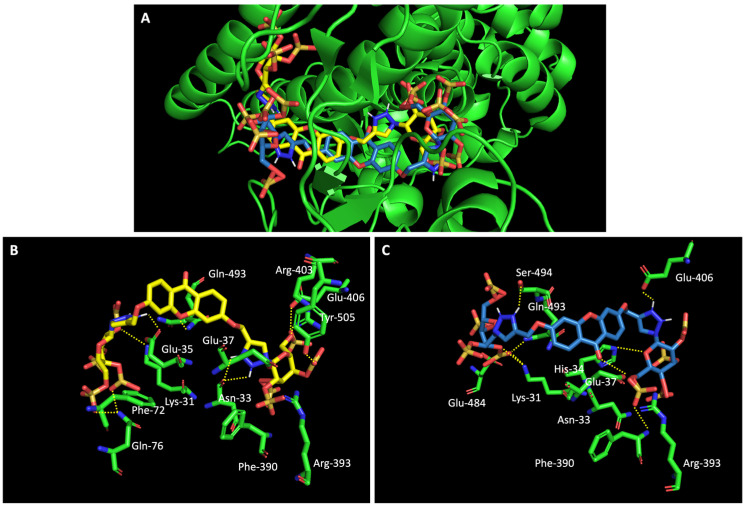
Molecular visualization of glucosulfated xanthone **7** and **9** in the RBD of Spike. (**A**) General view of compounds **7** (yellow) and **9** (blue). (**B**) Interaction between compound **7** and residues of the RBD of Spike. (**C**) Interaction between compound **9** and residues of the RBD of the spike protein. Polar interactions are represented as yellow dashes. Residues involved on those interactions are labeled: Arg—arginine; Asn—asparagine; Phe—phenylalanine; Gln—glutamine; Glu—glutamic acid; Gly—glycine; His—histidine; Lys—Lysine; Thr—threonine; Tyr—tyrosine.

When we examined the interaction of glucosulfated xanthones **7** and **9** with the target RBD of the spike protein (Figure 8), it shows that the derivatives bind differently, in opposite positions relative to one another. Despite that, both seem to share the binding of seven amino acids of the target, namely the polar amino acids Asn-33 and Gln-493, the negatively charged Glu-406 and Glu-37, the positively charged Arg-393 and Lys-31, and the amino acid Phe-390. The residue Gln-493 was previously described as being used in the interaction of the RBD of the spike protein and ACE2 [10]. 

### 2.4. Compounds ***1***, ***2***, and ***4*** Decreased the Production of New Virions and Their Release from Infected Vero CCL-81 Cells

Evaluation of viral titers was carried out in Vero CCL-81 cells inoculated with the supernatants of infected cells that had been previously treated with the IC_50_ of compounds **1**, **2**, **4**, **7**, and **9**, as previously described. Since the IC_50_ of **9** was not reached in the previous assay, a concentration of 50 μM was selected for this compound. The viral titer was determined by the number of FFU detected by the immunofluorescence staining for the SARS-CoV-2 spike protein. This reflects the number of new virions produced and released to the extracellular medium.

**Figure 9:**
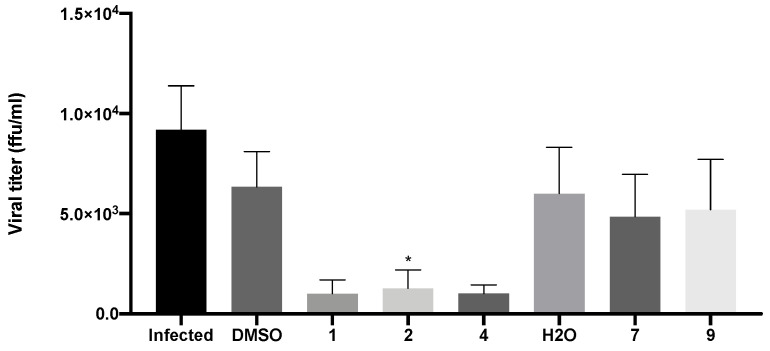
Viral titer measurement in Vero CCL-81 cells 48 h after inoculation with SARS-CoV-2 viral supernatants (obtained from cells previously treated with the IC_50_ of the tested compounds (or 50 μM for compound **9**) and then infected with SARS-CoV-2, MOI = 1). Infected cells without treatment were used as control. Data represents the mean ± SEM of at least three independent experiments. Analysis was performed by GraphPad using the Student *t*-test. * *p* < 0.05 relative to the vehicle (DMSO for compounds **1**, **2** and **4**; H_2_O for compounds **7** and **9**).

As expected, in the absence of treatment, there was production of new virions and their release from infected cells (9.6 × 10^3^ FFU/mL ± 5.2 × 10^3^; Figure 9). A decrease in viral titer was observed in Vero CCL-81 cells treated with both xanthenes **1** (1.4 × 10^3^ FFU/mL ± 1.4 × 10^3^) and **2** (1.7 × 10^3^ FFU/mL ± 2.0 × 10^3^), and bile acid derivative **4** (1.5 × 10^3^ FFU/mL ± 5.0 × 10^3^). Nonetheless, treatment with both glucosulfated xanthones **7** (6.1 × 10^3^ FFU/mL ± 4.2 × 10^3^) and **9** (7.0 × 10^3^ FFU/mL ± 4.6 × 10^3^) did not cause a significant alteration in the viral titer.

### 2.5. Compounds ***1***, ***2***, and ***4*** Significantly Decreased Spike Protein Levels in Infected Vero CCL-81 Cells, without Significantly Modifying ACE2 Levels

**Figure 10:**
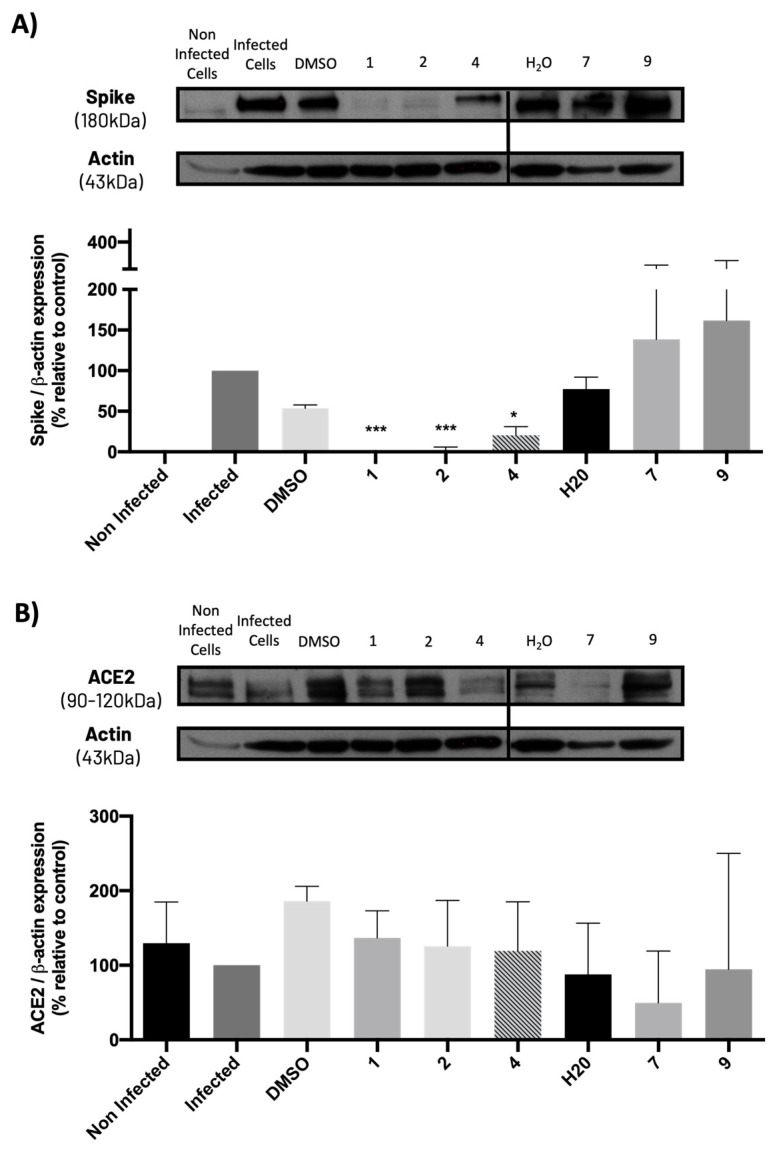
Levels of (**A**) spike and (**B**) ACE2 proteins in Vero CCL-81 cells treated with the IC_50_ concentrations of the tested compounds (or 50 μM for compound **9**) and infected with SARS-CoV-2 (MOI = 1), analyzed by Western blot. Actin was used as a loading control. Representative blots are shown. Data represents the mean ± SEM of at least three independent experiments. Analysis was performed by GraphPad using the Student *t*-test. * *p* < 0.05; *** *p* < 0.001 relative to the vehicle (DMSO for compounds **1**, **2** and **4**; H_2_O for compounds **7** and **9**).

The levels of the spike and ACE2 proteins on Vero cells previously treated with the compounds and subsequently infected with SARS-CoV-2 were also analyzed. Results (Figure 10A) show that non-infected cells do not present spike protein, while cells infected with SARS-CoV-2 exhibit this protein, as predicted. Additionally, compounds **1**, **2**, and **4** (at their IC_50_ concentration) significantly decreased the levels of spike protein compared to their vehicle (DMSO), while compounds **7** and **9** did not cause any significant change in spike protein levels when compared to their vehicle (H_2_O), which is in agreement with the previous results from the viral titration assay.

Moreover, results from Figure 10B show that ACE2 levels slightly decreased (although not significantly) in SARS-CoV-2-infected cells when compared to non-infected cells. Additionally, none of the compounds, particularly **1**, **2**, and **4**, significantly altered ACE2 levels when compared to the corresponding controls (vehicles: DMSO or H_2_O). Compound **7** caused a slight decrease in ACE2 levels, but this effect was not statistically significant.

### 2.6. Compounds ***1***, ***2***, ***7***, and ***9*** Did Not Exhibit Relevant Toxicity in Human Lung Cells

Since the previous studies were carried out in a non-human cell line, the effect of the most promising compounds (regarding viral titer reduction, **1**, **2**, and **4**) was assessed on ACE2 levels of an ACE2-expressing human cell line (MDA-MD-231). Treatment with 10 μM of these compounds for 48 h did not significantly affect ACE2 expression levels in MDA-MB-231 cells (Supplementary Figure S2). 

**Figure 11:**
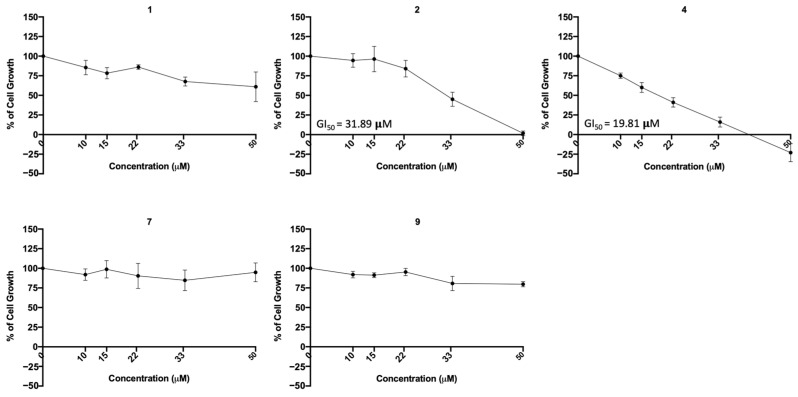
Concentration–response curve of the indicated compounds in the A549 human lung cell line. Cells were treated with five different concentrations (obtained by serial dilutions) of each compound for 48 h and analyzed by the SRB assay. Results are presented as percentage (%) of cell growth relative to the vehicle (DMSO for compounds **1**, **2** and **4**; H_2_O for compounds **7** and **9**). Data are expressed as mean ± SEM from three independent experiments.

To further investigate the possible cytotoxic effect of the most promising compounds (regarding viral infection inhibition, **1**, **2**, **4**, **7**, and **9**) in a human lung cell line, the sulforhodamine B (SRB) assay was performed in A549 cells treated with five serial dilutions of each compound for 48 h. The concentration–response curve as well as the GI_50_ concentration (concentration that inhibits 50% of cell growth) were determined. The results (Figure 11) indicate that compounds **1**, **7**, and **9** did not exhibited cytotoxicity at the tested concentrations, but compounds **2** and **4** presented cytotoxicity (GI_50_ = 31.89 μM and GI_50_ = 19.8 μM, respectively). 

## 3. Discussion

An increased affinity between ACE2 and the SARS-COV-2 spike protein is associated with higher infectivity of SARS-CoV-2 [20]. Therefore, we aimed to identify compounds from an in-house library with more than 300 small molecules, synthesized in the Laboratory of Organic and Pharmaceutical Chemistry from the Faculty of Pharmacy of the University of Porto, Portugal, with potential anti-SARS-CoV-2 activity by blocking the interaction between ACE2 and the spike protein. This library contains several bioactive compounds substituted with different functional groups, mainly sulfate groups and/or saccharic sulfated portions (GAG mimetics), but also sulfonamides, amines, amides, methoxy, and halogens, in order to explore other types of functional groups and understand the impact of non-GAG small molecule mimetics in the inhibition of ACE2–spike protein binding. 

From the structure-based virtual screening performed to identify small molecules with the potential to bind tightly to ACE2 and/or the spike RBD of SARS-CoV-2, ten promising compounds were selected for further functional assays. Upon analyzing the selected compounds, we observed that more than half are xanthones, which may be related to their highest number (142 xanthone derivatives) in our in-house library. Noteworthily, there are already studies pointing out the potential of xanthones as antiviral agents against SARS-CoV-2 [21,22,23]. Symmetry and bulky substituents seem to be important features for inhibition of the ACE2–spike interaction, as can be highlighted by the fact that five out of ten small molecules are symmetrical (**3**, **5**–**7**, and **9**) and the fact that six compounds present a molecular weight between 436.47 kDa and 488.59 kDa, while the remaining four have a molecular weight higher than 500 kDa. Some commercial drugs were also included in this in silico study, with the kinase inhibitor ponatinib presenting the best docking score. This result is in agreement with the literature, as kinase inhibitors have been shown to be potential antivirals, due to their action against key kinases required for viral entry, metabolism, or replication [24]. 

We next wanted to understand how treatment with the selected small molecules would affect the relative infection and viability of SARS-CoV-2-infected cells. The number of infected Vero CCL-81 cells decreased significantly in the presence of sulfonamide xanthenes **1** and **2**, which presented the lowest antiviral IC_50_. However, these two compounds exhibited cytotoxicity at the highest concentrations tested. The similar results obtained for these two sulfonamide xanthenes may be related to their chemical structure, which only differs in the presence of a methyl group. These results are consistent with those reported for the sulfonamide GLPG-0187 in a phase I clinical trial for the treatment of solid tumors, which was described as a potential blocker of SARS-CoV-2 infection in epithelial cells [25]. On the other hand, compounds **4**, **7** and **9** reduced viral infection without causing cytotoxicity in Vero CCL-81 cells. This effect for compound **4**, an amide derivative of deoxycholic acid, is in accordance with a recent positive clinical outcome following treatment with ursodeoxycholic bile acid after SARS-CoV-2 infection [26]. Compounds **7** and **9** are similar symmetrical triazol-linked glucosulfated xanthones obtained by click chemistry reaction that differ in the sulfated sugar substituent but share three main characteristics: (i) they are GAG mimetics, (ii) they present xanthone symmetry, and (iii) they have sulfated sugars linked by triazoles to the xanthone core. Glycan mimetics, such as fondaparinux (synthetic sulfated HS-binding pentasaccharide) and suramin (a small molecule mimetic of heparin) have previously been described as essential inhibitors of the early steps of SARS-CoV-2 infection [16,21]. Additionally, azole-containing small molecules have been studied for their potential in combating COVID-19. For example, itraconazole (a triazole antifungal agent) and elinexor (a triazole potential antineoplastic) have demonstrated potential anti-SARS-CoV-2 activity in vitro [27]. These results put in evidence the success of our virtual screening studies, since five of the ten synthetic small molecules selected by in silico studies were inhibitors of SARS-CoV-2 spike infection (**1**, **2**, **4**, **7** and **9**) and, thus, selected for further studies. 

Further in silico studies were accomplished for these five compounds to predict their possible binding to the targets. The results predicted several points of interaction, especially for glucosulfated xanthones **7** and **9**, which were visualized as interacting with residues of ACE2 described as being used by the RBD of the spike protein upon interaction with the host target [10,19]. Both compounds also seem to interact with residues of the RBD of SARS-CoV-2 that are supposedly essential in the interaction with ACE2 [10]. 

From the selected five compounds, only **1**, **2**, and **4** significantly reduced virions production in Vero CCL-81 cells and their release to the extracellular space, conditioning new infection in other cells. Thus, these compounds proved not only to efficiently inhibit viral entry, but also viral replication, raising the hypothesis that they might target additional molecules essential for the viral replication cycle. Additionally, these three compounds significantly reduced spike protein levels upon infection in Vero CCL-81 cells, in agreement with the previous results (on virion production). On the other hand, treatment with the glucosulfated xanthones **7** and **9** did not cause a significant alteration of the viral titer, nor in spike protein levels. Regarding treatment with xanthone **9**, we can hypothesize that the concentration used was not high enough, since the viral IC_50_ could not be determined in the previous assays. Thus, we can conclude that, at the tested concentrations, the glucosulfated xanthones **7** and **9** reduced viral entry but did not affect viral replication. 

The interaction between the spike protein and ACE2 has been implicated in SARS-CoV-2 pathogenesis and ability to infect cells [5], since a downregulation in ACE2 levels may lead to an aggravation of the disease [6]. Thus, promising results regarding cytotoxicity were obtained since hit compounds **1**, **2**, **4**, **7**, and **9** did not alter ACE2 expression significantly in Vero CCL-81 cells. 

The previous assays were conducted in a non-human cell line, raising the question of whether these compounds would be toxic to human cells. To address this concern, additional cytotoxic studies were conducted on two human cell lines: MDA-MB-231 (from human breast cancer) and A549 (derived from human lung cancer and commonly used in respiratory virus research). Since A549 cells do not express ACE2, the MDA-MB-231 cell line was used to study the effect of compounds on ACE2 expression in non-infected human cells. Additionally, A549 cells were used to assess their cytotoxicity in human lung cells. Our results show that both the bile acid derivative **4** and the sulfonamide xanthene **2** exhibited cytotoxicity (GI_50_ 19.81 μM and GI_50_ 31.89 μM). Nonetheless, the viral IC_50_ of xanthene **2** (11.91 μM) is lower than the GI_50_ concentration (31.89 µM) observed in the human A549 lung cells. Moreover, the results also put in evidence the low toxicity of the sulfonamide xanthene **1** and both glucosulfates triazole-linked xanthones **7** and **9**, by demonstrating that they are not toxic to human lung cells at their viral IC_50_, enhancing their potential as safe antiviral agents.

In conclusion and considering the summary of our results exhibited in Table 2, it is possible to observe that sulfonamide xanthenes (**1** and **2**) and bile acid derivative **4** displayed significant potential as anti-SARS-CoV-2 agents, effectively interfering with viral entry and replication. However, derivatives **2** and **4** exhibited cytotoxicity in human lung cells. On the other hand, glucosulfate triazole-linked xanthones (**7** and **9**) demonstrated promising results when reducing cell infection by SARS-CoV-2 (mostly compound **7**) but failed to inhibit viral replication. Importantly, our findings suggest that xanthene **1** holds significant potential for further development as an anti-SARS-CoV-2 agent, as it effectively inhibited both viral entry and replication, without exhibiting much cytotoxicity on human lung cells (the GI_50_ on these cells was not reached with the concentrations tested).

Future work will further explore the cytotoxicity and anti-SARS-CoV-2 activity of these compounds in human lung cells that express ACE2 and the toxicity of the compounds (including cardiotoxicity) will be confirmed by other assays. Additional studies will also be conducted to better elucidate the mechanism of action of these compounds, as well as to synthesize and investigate the activity of structurally related derivatives in order to establish structure–activity relationships and identify essential substituents for anti-SARS-CoV-2 activity.

## 4. Materials and Methods

### 4.1. Cell Lines and Culture Conditions

Vero CCL-81 cells from the kidney of the African green monkey, A549 cells from human lung cancer and MDA-MB-231 cells from human breast cancer used in this work were purchased from the American Type Culture Collection (ATCC). The cell lines Vero CCL-81 and A549 were cultured in Dulbecco’s Modified Eagle Medium (DMEM; Thermo Scientific, Waltham, MA, USA) supplemented with 10% fetal bovine serum (FBS; Biowest, Nuaillé, France; S181H-500) and MDA-MB-231 cells were maintained in Roswell Park Memorial Institute (RPMI-1640) medium supplemented with Stable Glutamine and 25 mM HEPES (Lonza, #BE12-115F/U1), complemented with 10% of FBS. For the SRB assay and infection assays, cells were grown in medium supplemented with 5% FBS. Cells were cultured in tissue culture flasks and kept at 37 °C in a humidified chamber containing 5% CO_2_. Cells were then observed using an inverted light microscope (Leica DMi1, Leica Biosystems, Wetzlar, Germany). Cells were genotyped and tested for mycoplasma infection. All experiments were carried out with cells at the exponential growth phase and with more than 90% viability. 

### 4.2. Virus 

SARS-CoV-2 was previously isolated from a positive nasopharyngeal sample diagnosed in a Portuguese individual in January 2021. The virus was stored at −80 °C prior to virus isolation. RT-PCR has been previously used to confirm the molecular diagnosis for three SARS-CoV-2 genes (ORF1ab, E and N—Fosun COVID-19 RT-PCR Detection Kit; Fosun Pharma USA Inc., Princeton, NJ, USA). The isolation had been previously performed by inoculation in Vero cells, and the viral sequencing had been previously performed by next-generation sequencing, as previously described [28], allowing to confirm that it could be affiliated to B.1.1.7 variant, according to PANGO nomenclature, or alpha variant according to the OMS classification. 

In accordance with institutional guidelines, all experiments involving infectious SARS-CoV-2 were conducted in biosafety level 3 (BSL3) conditions. 

### 4.3. Molecular Docking Virtual Screening

The crystal structures of ACE2 (PDB code 6M18) [15] and SARS-CoV-2 RBD of spike (PDB code 6M0J) [10] were obtained from the Protein Data Bank (PDB) [29]. The following steps were followed in the preparation of the target protein using AutoDockTools (Scripps Research, La Jolla, CA, USA): (i) removal of all water molecules and ATP, (ii) addition of hydrogen atoms, (iii) and calculation of atomic partial charges. 

The in-house compounds, the known drugs (darunavir, lopinavir, ponatinib, remdesivir, and ribavirin), and the positive controls of SARS-CoV-2 peptide (C480-C488) [10] and all-trans retinoic acid [30] were drawn using ChemDraw 16.0 (PerkinElmer Informatics, MA, USA). Minimization was carried out using the Austin Model 1 parameterization of the MNDO method (AM1) implemented in ArgusLab 4.0.1. The calculation proceeded until the gradient between consecutive steps in the geometry search was inferior to 0.1 kcalA^−1^mol^−1^. When chirality existed, it was preserved. Partial charges were calculated following the standard parameters of the force field. For docking, AutoDock Vina (Scripps, CA, USA) was employed [31]. The center coordinates were set as 170.90, 115.25, 243.90, and −36.40, 29.43, 3.32 for ACE2 and the spike protein of RBD, respectively. The grid box size was set to 37 × 31 × 35 and 18 × 38 × 20 grid points for ACE2 and the spike protein of RBD, respectively. Exhaustiveness was set to 8. Results were ranked based on their docking score (kcal/mol). 

### 4.4. Tested Compounds

Ponatinib was purchased from Quimigen (AP24534). The synthesis of the following compounds were previously described: **1**–**3** [32]; **6**, **7**, and **9** [33]; **8** [34]; **4**, **5**, and **10** will be published elsewhere. The compounds were prepared as 60 mM stock solution in dimethylsufoxide (DMSO; Merck Life Science, Darmstadt, Germany; D2650) or in sterile water for Molecular Biology grade (Merck Life Science, Darmstadt, Germany; 95284), and stored at −20 °C. 

### 4.5. Effect of the Compounds in Cell Lines

#### 4.5.1. In Non-Infected Cell Lines

To evaluate the effect of the tested compounds on cell viability, in the absence of viral infection, Vero CCL-81 cells were seeded 24 h prior to compound addition in 96-well imaging plates, at a density of 1 × 10^4^ per well, and cultured overnight at 37 °C in 5% CO_2_. Five final concentrations (10.00 μM, 14.96 μM, 22.36 μM, 33.44 μM, and 50.00 μM) of each compound resuspended in DMEM containing 5% FBS were tested in the seeded cells (in duplicate), by replacing the culture medium. DMSO was used as control. After 48 h incubation with the compounds, cells were fixed, permeabilized, and stained for 30 min for nuclear markers, with DAPI (Thermo Fisher, Waltham, MA, USA), and for cell delineation markers, with HCS CellMask (Thermo Fisher, Waltham, MA, USA). In the end, images of each well were acquired on the IN Cell Analyzer 2000, using a 10× magnifying objective. Image analysis was performed by using image segmentation and the quantification software “Developer Toolbox” (version 1.9.3, x64, GE Healthcare, Chicago, IL, USA) and CellProfiler [35]. DAPI was used to assess the number of cells. Three independent experiments were performed. Data were presented as percent compared with control wells (“blank”, non-infected cells without the presence of the compounds). The IC_50_ values in the absence of infection were calculated with Prism 9 software.

#### 4.5.2. In Infected Cell Lines with SARS-CoV-2

To evaluate the effect of the small molecules on viral inhibition, Vero CCL-81 cells infected with SARS-Cov-2 were used. The procedure was identical to the evaluation of cell viability in the absence of infection, except for the following points: (i) 1 h after the addition of the compounds, the plates were transferred to the BSL3 facility and infected with SARS-CoV-2 by adding 50 μL of inoculum to each well, with a final MOl of 1; (ii) after 48 h in the presence of compounds and viruses, cells were treated as previously described, but were also fixed and incubated with mouse anti-SARS-CoV-2 spike antibody (GTX632604; GeneTex, Irvine, CA, USA). The output of infection was measured as the fluorescence of the viral protein stained with Texas Red, and DAPI was used to assess the number of cells, as previously described. The non-infected and the infected cells in the absence of treatments were included as controls. Three independent experiments were performed, and data were presented as percentage compared with control wells (“blank”, infected cells without the presence of the compounds). The IC_50_ values for each tested compound in the presence of infection were calculated with Prism software.

### 4.6. Cytotoxicity Using the Sulforhodamine B (SRB) Assay

To determine the cytotoxic effect of the tested compounds, the SRB assay was performed according to the previously described protocols [36,37]. The concentration of each compound that caused 50% of cell growth inhibition (GI_50_) was determined from the dose response curve. A549 cells were plated in 96-well plates at a previously determined optimal cell concentration (5 × 10^4^ cells/mL) and incubated for 24 h. Then, cells were treated with different concentrations of the tested compounds for 48 h. The following procedure was accomplished as previously described [36,37]. Using the Gen5^TM^ software (version 1.04.5), the absorbance was measured at 510 nm in a multiplate reader (Synergy^TM^ Mx, Biotek Instruments Inc., Winooski, VT, USA). 

### 4.7. Protein Expression Analysis by Western Blotting

#### 4.7.1. In Non-Infected Cell Lines

For protein expression analysis, MDA-MB-231 and Vero CCL-81 cells were seeded in 6-well plates and incubated for 24 h. Then, the cells were treated with the tested compounds at 10 μM or at their respective IC_50_. Cell pellets were collected after 48 h incubation, and the following lysis, quantification, and Western Blot procedures were performed as previously described [36], except for the following points: (i) no phosphatase inhibitor was used; (ii) a total of 15 μg of protein lysates obtained from MDA-MB-231 cells treatment or 50 μg of protein lysates obtained from Vero CCL-81 cells were loaded and separated in 10% SDS-Page gels; (iii) the transference to a nitrocellulose membrane (GE Healthcare Life science, Chalfont St Giles, UK; GE10600002) occurred for 1 h 45 min at 100 V; (iv) the primary antibodies (1:2000) used were: mouse anti-ACE2 monoclonal antibody (CL4035; Thermo Fischer Scientific; Waltham, MA, USA) and mouse anti-actin (sc-47778; Santa Cruz Biotechnology, Dallas, TX, USA). 

#### 4.7.2. In Cell Lines Infected with SARS-CoV-2

For protein expression analysis of infected cells, Vero CCL-81 cells were seeded in 6-well plates and incubated for 24 h. The procedure was identical to the protein extraction in the absence of infection, except for the following points: (i) 1 h after the addition of the compounds, the plates were transferred to the BSL3 facility and infected with SARS-CoV-2 by adding 100 μL of inoculum to each well, with a final MOl of 1; (ii) after 48 h in the presence of compounds and viruses, supernatants were collected for viral titration and cell pellets were washed with PBS by centrifugation at 1000 rpm for 5 min at 4 °C, and lysed in Wynman’s buffer. Then, cell lysates were transferred again to BSL1 to proceed with protein extraction and Western blot, as described above, except for the following point: membranes were first incubated with the primary antibody (1:2000): mouse anti-SARS-CoV-2 spike antibody (GTX632604; GeneTex, Irvine, CA, USA). After incubation with the secondary antibody, washing steps and detection (as described above), a mild stripping step was carried out by washing the membranes with TBS-T for 15 min at RT, adding a stripping solution (10% MeOH and 10% acetic acid in water) for 15 min at RT, and then washing again with TBS-T. Membranes were then blocked and incubated with primary antibodies (1:2000): mouse anti-ACE2 monoclonal antibody (CL4035; Thermo Fischer Scientific; Waltham, MA, USA) and mouse anti-actin (sc-47778; Santa Cruz Biotechnology, Dallas, TX, USA), as mentioned earlier. 

### 4.8. Viral Titration

Viral titration was determined using the focus forming assay (FFA). The procedure was followed as previously described [28,38]. 

### 4.9. Statistical Analyses

Statistical analyses were conducted using GraphPad Prism V9.0 software. Student’s *t*-test was used for data analysis. *p* values of <0.05 were considered statistically significant. Data are presented as mean ± standard error of mean (SEM) for a minimum of three independent experiments. 

## Figures and Tables

**Table 1:** Docking score of the ten compounds selected from our in-house library, and ponatinib, for the ACE2 binding pocket (6M18) and the RBD of the spike protein (6M0J), calculated using Autodock Vina.

Code	Structure	Docking Score ACE2 (kcal/mol)	Docking Score RBD of Spike (kcal/mol)
1	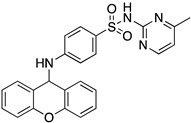	−8.7	−9.0
2	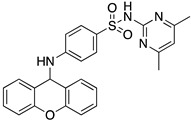	−8.9	−9.1
3	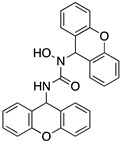	−8.7	−7.8
4	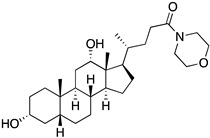	−8.9	−7.4
5	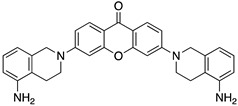	−9.8	−9.4
6	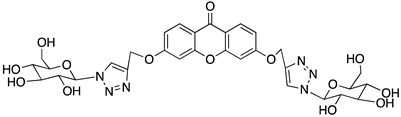	−9.4	−9.4
7	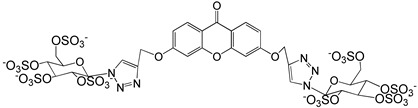	−9.3	−9.0
8	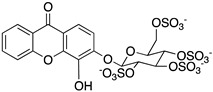	−9.1	−8.7
9	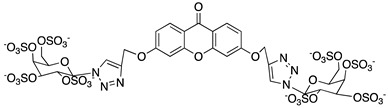	−8.7	−7.8
10	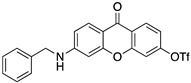	−8.3	−9.1
Ponatinib	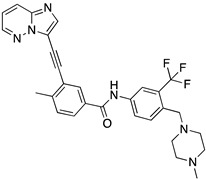	−8.3	−8.1

**Table 2:** Summary of the results obtained for compounds **1**, **2**, **4**, **7**, and **9**.

Compounds	Antiviral IC_50_ (Vero CCL-81 Monkey Cells)	Cytotoxic IC_50_ (Vero CCL-81 Monkey Cells)	Viral Titer and Spike Levels (Vero CCL-81 Monkey Cells)	ACE2 Levels (Vero CCL-81 Monkey Cells)	Cytotoxic IC_50_ (A549 Human Lung Cells)
**1**	12.62 μM	18.43 μM	↓↓	−	n.d.
**2**	11.91 μM	17.22 μM	↓↓	−	31.89 μM
**4**	24.07 μM	n.d.	↓↓	−	19.81 μM
**7**	31.69 μM	n.d.	−	−	n.d.
**9**	n.d.	n.d.	−	−	n.d.

n.d., not determined in the concentration range tested; ↓↓ significant reduction; −, not significantly altered.

## Data Availability

Not applicable.

## Supplementary Materials

The following supporting information can be downloaded at: https://www.mdpi.com/article/10.3390/molecules28207204/s1, Figure S1: Effect of the selected compounds on Vero CCL-81 cells and on Vero CCL-81 cells further infected with SARS-CoV-2 (MOI = 1). Figure S2: Expression levels of ACE2 in MDA-MB-231 cells treated with 10 μM of the compounds **1**, **2**, and **4** for 48 h, analyzed by Western Blot.

## Sample Availability

Samples of the compounds **1**–**10** are available from the authors.
