# Supplementary material for: Evaluation of the Cytotoxic and Antiviral Effects of Small Molecules Selected by In Silico Studies as Inhibitors of SARS-CoV-2 Cell Entry
Source: Molecules. 2023 Oct 21;28(20):7204. doi: 10.3390/molecules28207204 (PMC10609270; doi:10.3390/molecules28207204)
Supplement: Supplementary file 1 [file molecules-28-07204-s001.zip › molecules-2637564-supplementary.pdf]

Supplementary material

Evaluation of cytotoxic and antiviral effects of small molecules selected by in silico studies as inhibitors of SARS-CoV-2 cell entry

Francisca Carvalhal[1,2,3,4], Ana Cristina Magalhães[3,4], Rita Rebelo[1,3,4], Andreia Palmeira[1,2], Diana I. S. P. Resende[1,2], Fernando Durães[1,2], Miguel Maia[1,2], Cristina P.R. Xavier[3,4], Luísa Pereira[3,4], Emília Sousa[1,2], Marta Correia-da-Silva[1,2*] and M. Helena Vasconcelos[1,3,4*]

1 FFUP—Faculty of Pharmacy, University of Porto, 4050-313 Porto, Portugal; fcarvalhal@i3s.up.pt (F.C.); rrebelo@ipatimup.pt (R.R.); apalmeira@ff.up.pt (A.P.); dresende@ff.up.pt (D.I.S.P.R.); fduraes5@gmail.com (F.D.); miguelmaia2@gmail.com (M.M.); esousa@ff.up.pt (E.S.)

2 CIIMAR—Interdisciplinary Centre of Marine and Environmental Research, 4408-208 Matosinhos, Portugal

3 i3S—Instituto de Investigação e Inovação em Saúde, University of Porto, 4200-135 Porto, Portugal; acmagalhaes@i3s.up.pt (A.C.M.); cristinax@ipatimup.pt (C.P.R.X.); luisap@i3s.up.pt (L.P.)

4 IPATIMUP—Institute of Molecular Pathology and Immunology, University of Porto, 4200-135 Porto, Portugal

* Correspondence: m_correiadasilva@ff.up.pt (M.C.-d.-S.); hvasconcelos@ipatimup.pt (M.H.V.)

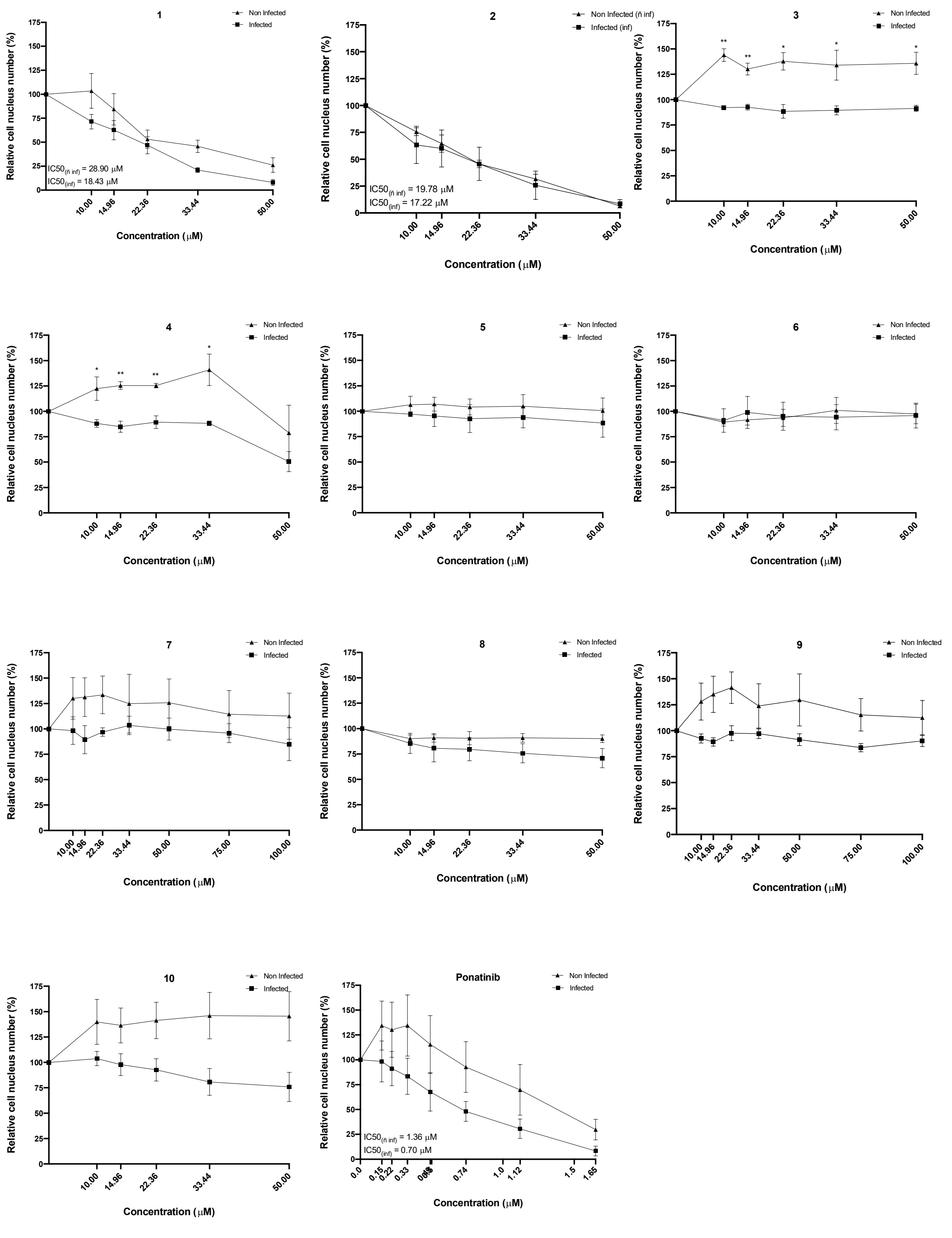

Supplementary Figure S1. Effect of the selected compounds on Vero CCL-81 cells and on Vero CCL-81 cells further infected with SARS-CoV-2 (MOI=1). Non-infected cells were treated with the compounds at the indicated concentrations for 48 h (non-infected, lines with triangles). Alternatively, Vero CCL-81 cells were treated with the compounds at the indicated concentrations and further infected with SARS-CoV-2 for 48 h (infected, lines with squares). After fixation with 4 % PFA, all cells were stained with DAPI and analyzed using the IN Cell Analyzer 2000. Plots represent the concentration–response curve for each compound regarding cytotoxicity based on cell nuclei number compared to the appropriate control (non-infected cells without treatment for non-infected cells, and infected cells without treatment for infected cells). Values

represent the mean ± SEM of three independent experiments (each experiment performed in duplicate). Student t-test was performed by GraphPad. *p < 0.05; **p < 0.01 relative to the control (non-infected cells with treatment).

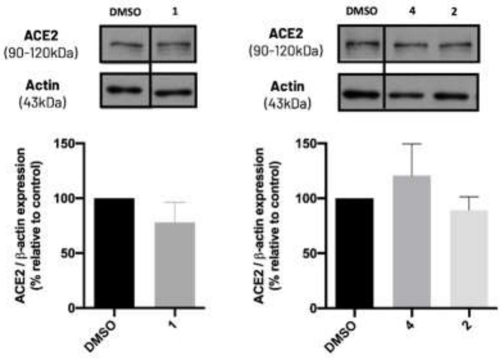

Supplementary Figure S2. Expression levels of ACE2 in MDA-MB-231 cells treated with 10 μM of the compounds **1**, **2** and **4** for 48 h, analyzed by Western Blot. Actin was used as a loading control. Representative blots are shown. Results are the mean ± SEM of three independent experiments.
